# Successful closure of a large abdominal wall defect using endoscopic component separation technique in an infant with a giant ventral hernia after staged surgery for omphalocele

**DOI:** 10.1186/s40792-020-01087-2

**Published:** 2021-01-06

**Authors:** Miki Toma, Toshihiro Yanai, Shiho Yoshida

**Affiliations:** Department of Pediatric Surgery, Ibaraki Children’s Hospital, 3-3-1, Futabadai, Mito, Ibaraki 311-4145 Japan

**Keywords:** Abdominal wall defect, Ventral hernia, Endoscopic, Component separation technique, Infant

## Abstract

**Background:**

The management of large abdominal wall defects, such as omphalocele or gastroschisis, remains a challenge for pediatric surgeons. Though several techniques have been described to repair those conditions, there is no procedure considered to be the standard worldwide. We report an infant girl with a giant ventral hernia after staged surgery for omphalocele in whom delayed closure of a large abdominal wall defect was performed successfully using “endoscopic component separation technique (ECST)” without serious complications.

**Case presentation:**

A baby girl was admitted to our hospital because of a giant omphalocele, which had been prenatally diagnosed. The omphalocele was supraumbilical and included the entire liver. After staged surgery, a large abdominal wall defect was closed by skin, creating a giant ventral hernia. We performed endoscopic separation component technique (ECST) for the closure of her abdominal wall defect when she was 11 months of age. ECST was initiated with placement of a 5.0-mm port just above the inguinal ligament and under the external oblique muscle. The space between the external and internal oblique muscles was created by the insufflation pressure, and a second 5.0-mm port was placed at 1.0 cm below the inferior edge of the rib into the space. As the further dissection was carried, the aponeurosis of the external oblique muscle was identified as a white line, running vertically from the epigastrium to inguinal ligament. It was transected longitudinally using electrocautery over its full length. The same procedure was performed on the contralateral side and the abdominal wall was successfully closed. Postoperative course was uneventful.

**Conclusions:**

The technique of ECST, described here, is simple and safe for infants, and the cosmetic result is satisfactory.

## Background

The management of large abdominal wall defects, such as omphalocele or gastroschisis, poses an immense challenge for pediatric surgeons. Despite the application of several strategies and techniques, there are, to the best of our knowledge, no studies showing the superiority of any of the techniques over others.

Since Ramirez et al. [1] reported his first three cases using component separation techniques (CST) to repair ventral hernias in 1990, this technique has been widely used in adult patients. CST is based on enlargement of the abdominal wall surface by translation of the muscular layers without using prosthetic material and without compromising the innervation of and blood supply to the muscles. In 2008, van Eijck et al. [2] demonstrated the effectiveness of CST for secondary closure in children with a giant abdominal wall defect as well.

Later, endoscopic CST (ECST) has developed as preferable procedure resulting in decreasing skin complications compared to the original CST, but there has been no report in which ECST was used to treat a giant ventral hernia in children. Here, we present a case of an infant with a large abdominal wall defect that was successfully repaired using ECST.

## Case report

The patient was prenatally diagnosed with omphalocele at 30 weeks of gestation. Because of the giant omphalocele, the female infant was delivered by a planned cesarean section at 38 weeks. Her Apgar score was 7 at 1 min, and birth weight was 3107 g. The giant omphalocele, measured 7.0 cm in diameter, was supraumbilical, and included the entire liver. Except for patent ductus arteriosus, no other intracardiac anomalies were detected. At birth, the omphalocele was covered using a wound retractor as a silo, which was anchored on the edge of the abdominal skin. Each day, the skin was gradually retracted, until the height of the omphalocele reached the level of skin. On day 7, she was performed for closing the abdominal wall. The entire herniated liver was retracted medially, revealing a giant right diaphragmatic hernia, which had not recognized until second laparotomy. The entire small intestine and a portion of the colon were drawn out of the thoracic cavity, and an expanded polytetrafluoroethylene surgical membrane (Gore-tex® ePTFE patch) was applied repairing the entire defect of the right diaphragm. The viscera could not be placed into the abdominal cavity due to the increased intra-abdominal pressure, therefore, a wound retractor (a silo) was applied on the edge of the abdominal defect, and the abdominal skin closure was performed on day 20, creating a giant ventral hernia. The closure of the large abdominal wall defect by ECST was planned when the infant was 11 months of age and had attained 7.0 kg of body weight.

### Technique

**Fig. 1:**
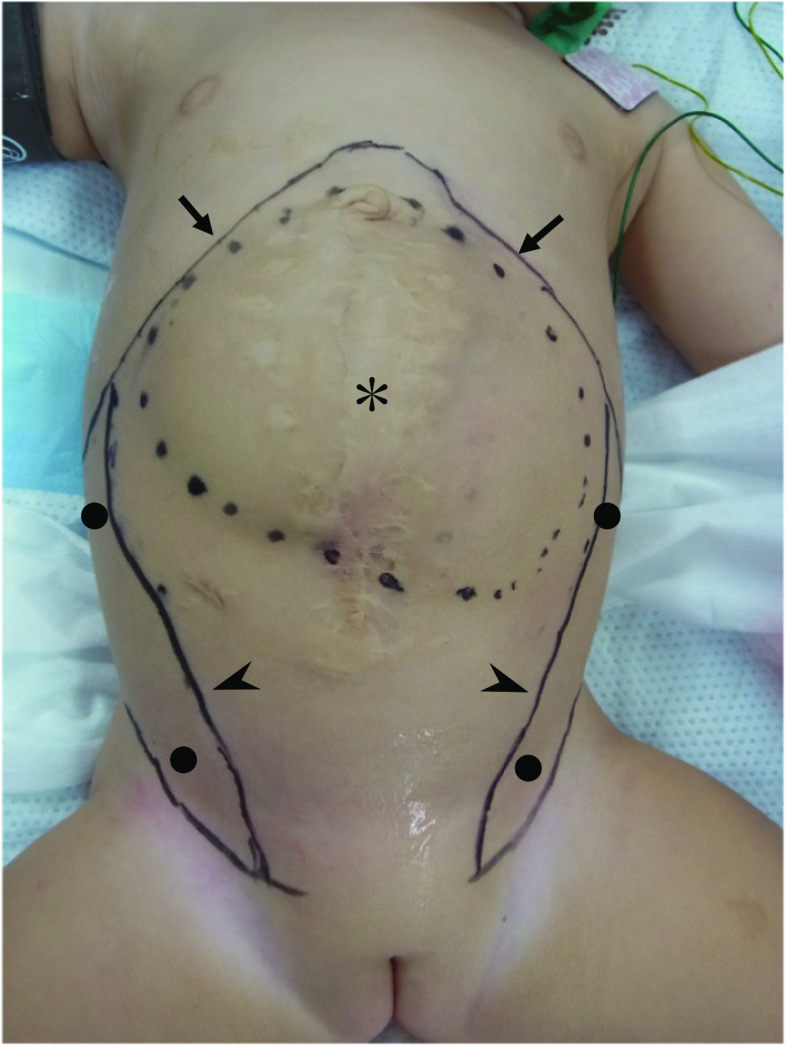
Preoperative appearance of the patient with protruded liver covered by the skin only ($\ast$). The inferior edges of the rib are marked by arrows ( →), and the lateral edges of the rectus muscles are marked by arrowheads (➤). Port sites (●) are at subcostal and inguinal regions on both sides

The patient received preoperative bowel preparation, urethral catheterization and preoperative antibiotics, following which she was placed in the supine position with her arms stabilized upward. The inferior edge of the rib and the lateral edge of the rectus abdominis muscle were marked under ultrasound guidance (Fig. 1). The procedure was initiated by first thoroughly sterilizing the abdomen, followed by reopening of the prior midline incision and entire dissection of the skin from the protruded liver surface until the medial edge of the rectus abdominis muscle was identified. This maneuver took the longest time, because the adhesion between the skin and liver was remarkable.

**Fig. 2:**
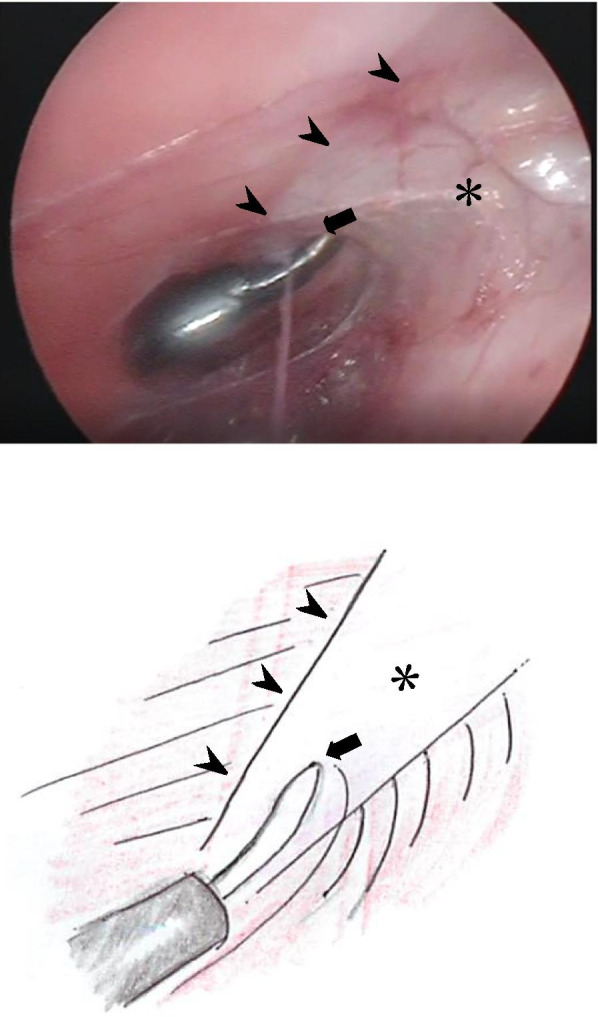
Endoscopic view and a schema of the space between the external and internal oblique muscles. The aponeurosis ($\ast$) was identified as the white line, just medial to the external oblique muscle (➤) and running vertically from the epigastrium to the inguinal ligament. Transection of the aponeurosis was carried by the electrocautery (➡)

**Fig. 3:**
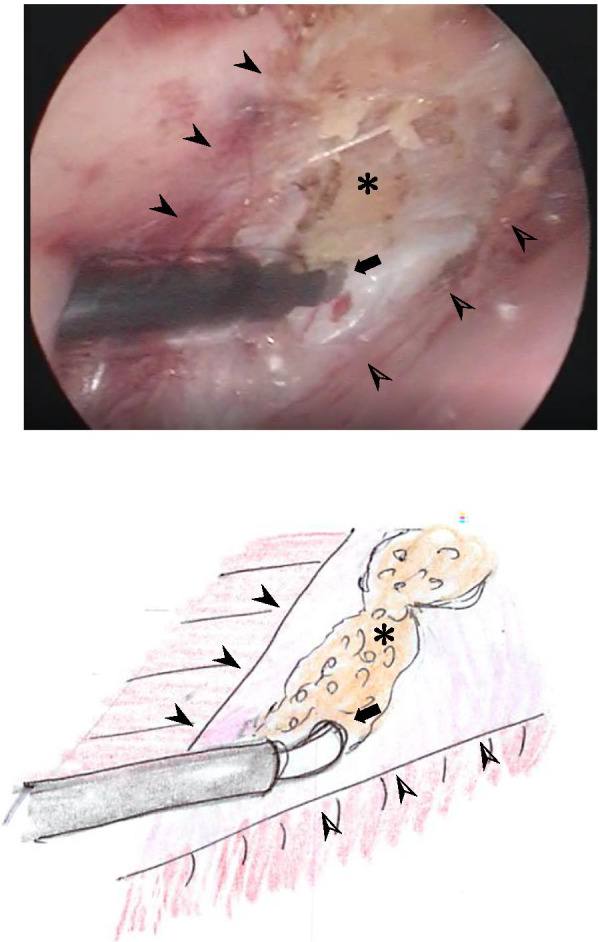
Endoscopic view and a schema of the aponeurosis being transected by the electrocautery (➡). The subcutaneous fat ($\ast$) is visible between the rim of the external oblique muscle (➤) and the rectus abdominis muscle (➣)

**Fig. 4:**
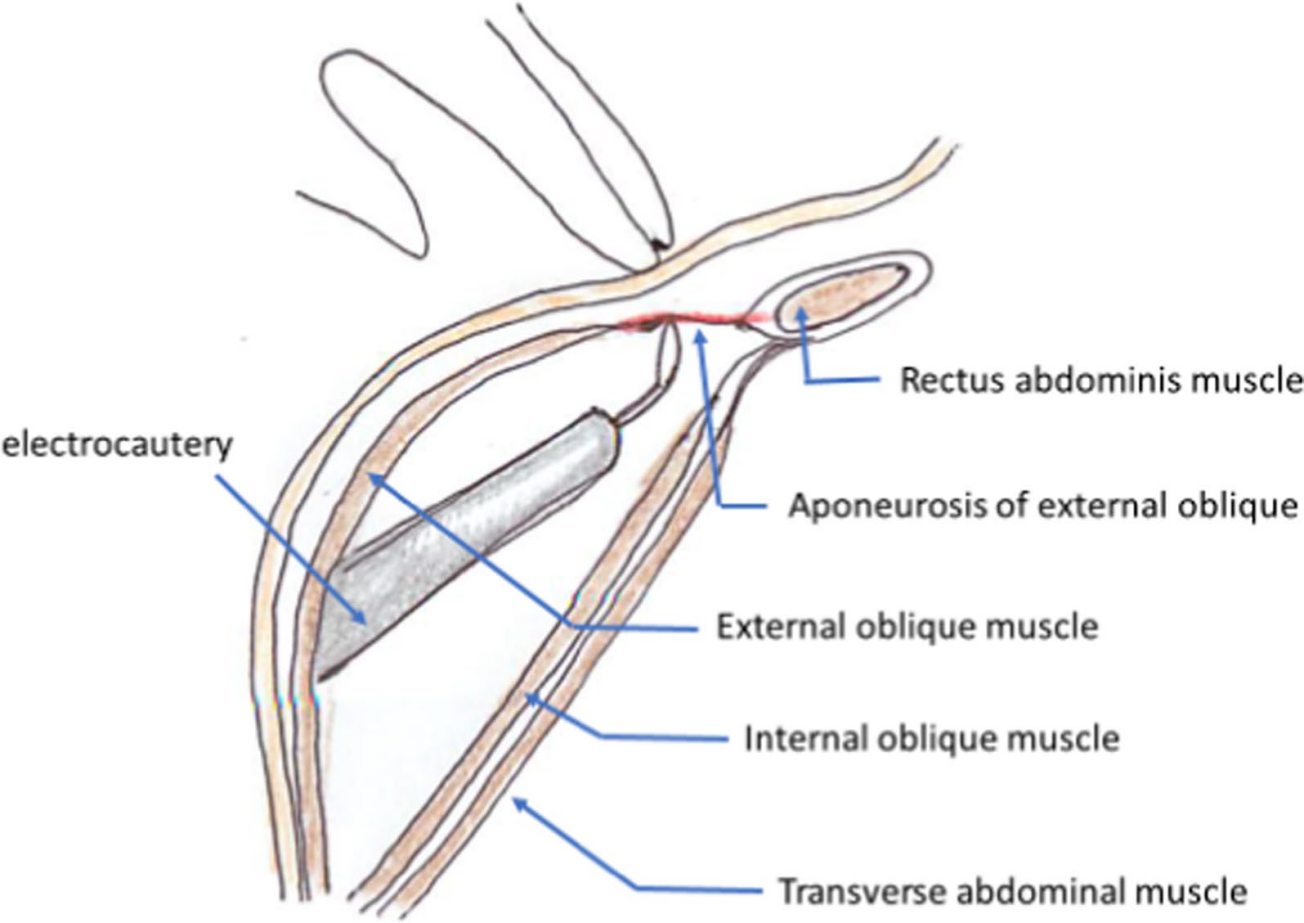
A schema of the procedure. Electrocautery was inserted into the space between the external and internal oblique muscles, and the aponeurosis of the external oblique muscle was transected by a slight touch of electrocautery. The skin above was lightly pushed by surgeon’s finger which makes the aponeurosis stretched and transected easily

ECST was initiated with a small incision just above the inguinal ligament lateral to the rectus, and a 5.0 mm port was placed under the external oblique muscle (Fig. 1). The space between the external and internal oblique muscles was created by carbon oxide insufflation at pressures ranging from 6 to 10 mmHg and blunt dissection using a 30° laparoscope under direct vision. A second 5.0-mm port was placed at 1.0 cm below the inferior edge of the rib into the space created (Fig. 1) and further dissection was performed until the aponeurosis of the external oblique muscle, appearing as a white line, running vertically from the epigastrium to inguinal ligament, was clearly visible (Fig. 2). Then the aponeurosis was transected longitudinally using electrocautery over its full length (Fig. 3). While transecting aponeurosis, we lightly pushed the skin down in order to transect aponeurosis much easily (Fig. 4). The width of subcutaneous space between the rectus abdominis and the external oblique muscle was subsequently widened as the transection proceeded and the external oblique muscle was retracted laterally. The same procedure was performed on the contralateral side.

**Fig. 5:**
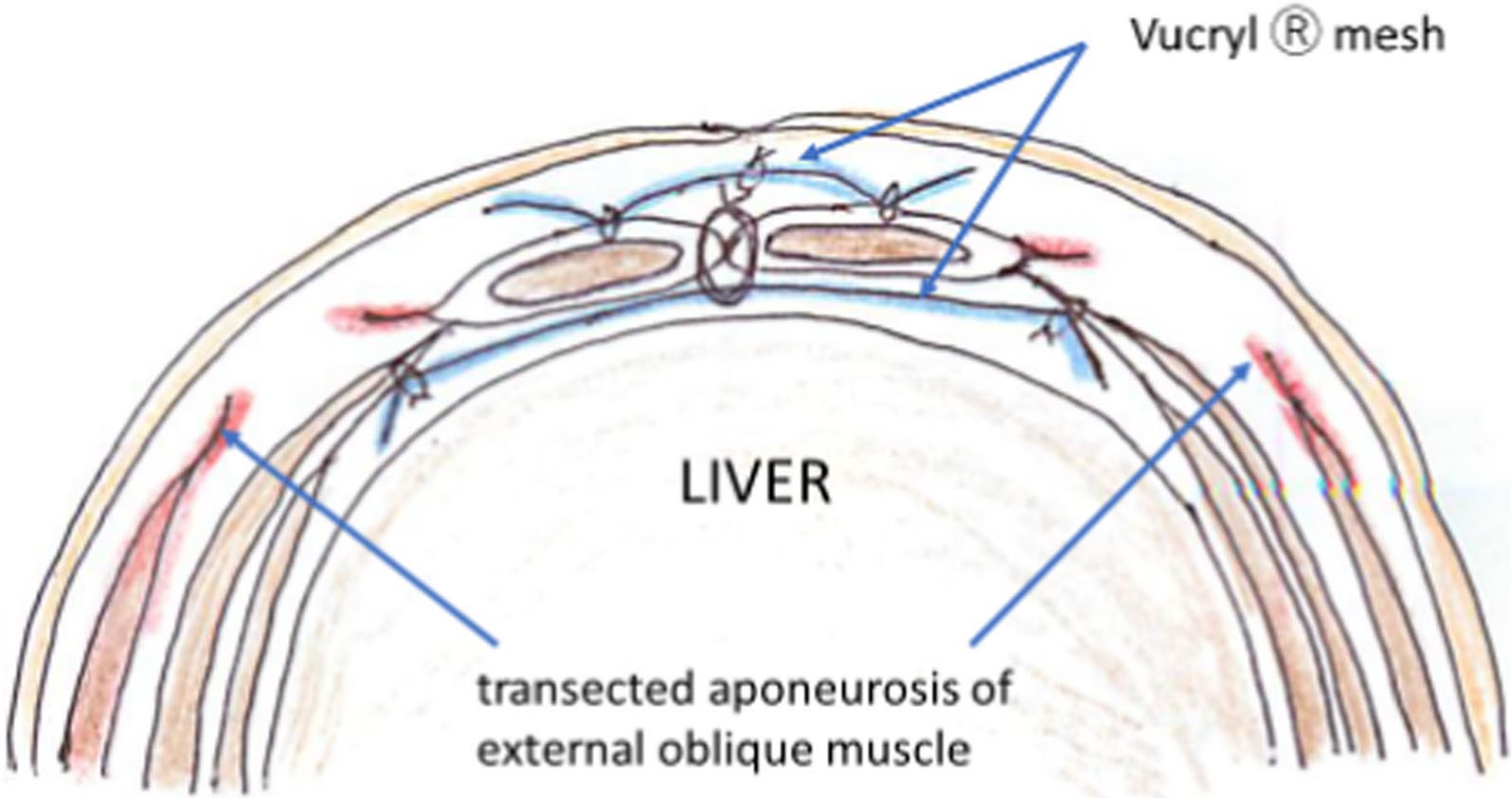
A schema of the abdominal wall after the procedure. Underlay Vicryl^Ⓡ^ mesh was fixed to the transversalis fascia and onlay mesh was fixed to the rectus abdominis muscle

**Fig. 6:**
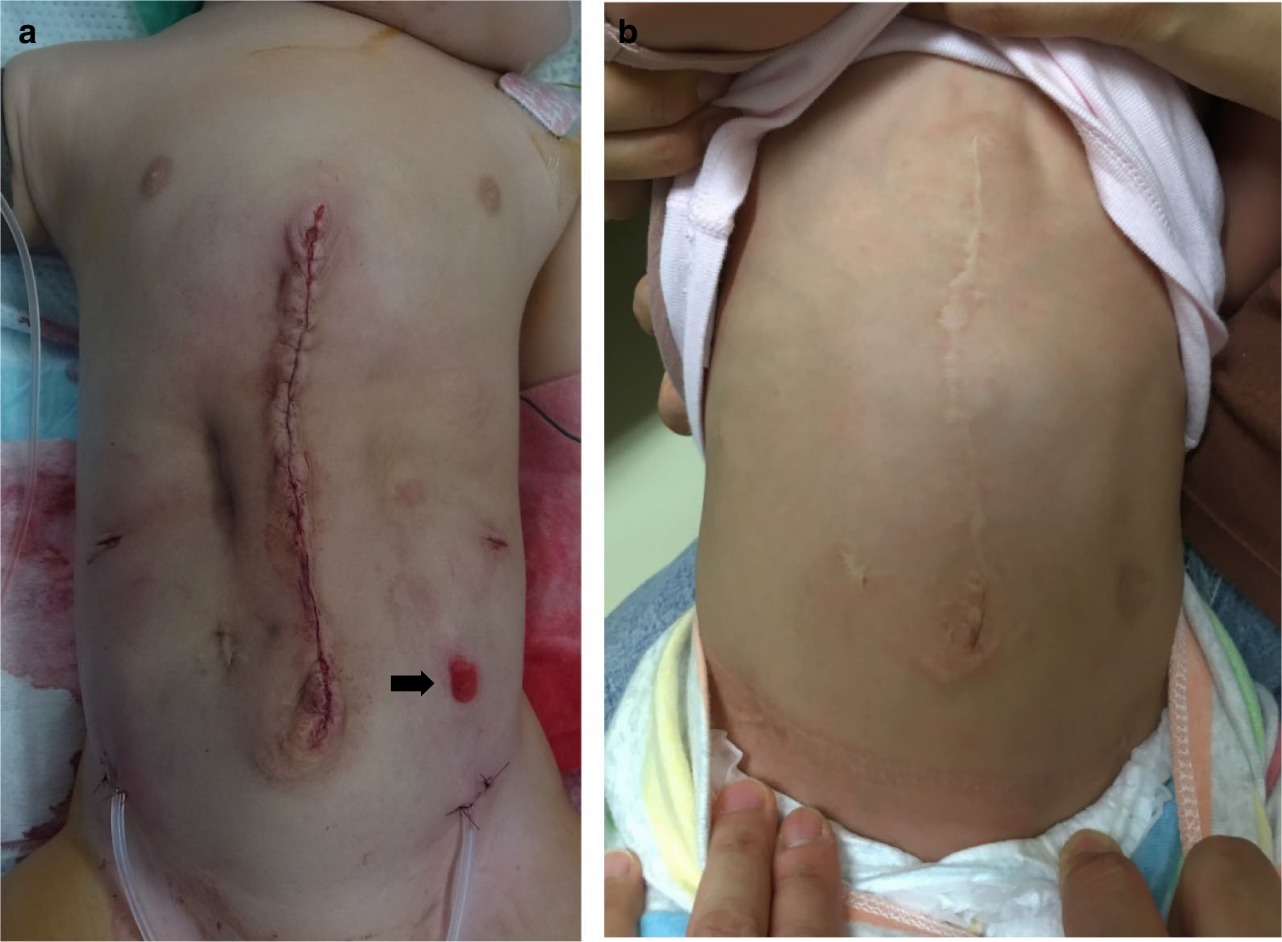
**a**: Appearance of the patient just after ESCT, a small skin burn (➡) occurred on the left abdomen. Drains were placed in the created spaces from the inguinal wounds.** b**: Appearance of the patient at 4 years of age

Upon transection, both medial edges of the rectus abdominis muscles were approximated with slight tension. A Vicryl® mesh was placed in onlay and underlay positions with respect to the rectus abdominis muscle to reduce tension (Fig. 5). The surgery was completed in 535 min (CO_2_ insufflation time was 88 min, total amount of blood loss was 40 ml and without blood transfusion). Appearance of the patient after ECST was shown in Fig. 6a. A complicated small burn occurred on the skin caused by electrocautery during the transection of the aponeurosis. The patient was kept sedated for 5 days, during which a muscle relaxant was administered for the first 3 days to reduce tension of the medial wound. Enteral feeding was started on 5th postoperative day and oral intake was on 9th postoperative day. The patient was discharged on 16th postoperative day. At the time of this report, the patient was 4 years of age, and she exhibited a satisfactory and normally shaped abdomen (Fig. 6b).

## Discussion

Management of giant omphaloceles remains a challenge for pediatric surgeons because of the restricted abdominal cavity and large abdominal wall defects. Because single-stage procedures may lead to a high abdominal pressure, resulting in multiple organ failure and respiratory impairment, primary skin closure or epithelization of omphaloceles followed by delayed closure of the abdominal wall has become the mainstay of treatment. Various delayed closure techniques have been reported, but no technique has demonstrated advantages over other techniques. CST, first reported by Ramirez et al. [1], has become a preferred technique for giant incisional hernia without using prosthetic materials in adults.

This technique is unique in that the functional transfer of the abdominal musculature provides stable and dynamic support to the abdominal wall. It was first used to a pediatric case for the delayed repair of a giant omphalocele by Wijnen et al. [3] and followed by van Eijck [2], who reported 10 consecutive cases. Subsequently, Levy et al. [4] demonstrated that CST can be used for the closure of primary defects in newborns. However, the frequency of skin complications, including skin necrosis and wound dehiscence, has increased to up to 30–50% [2, 5–7] in CST because of the mandatory need for wide dissection of the subcutaneous tissue, resulting in interruption of blood supply to the abdominal wall.

Lowe et al. [5] first reported ECST as preferable procedure. In ECST, the wide dissection of the subcutaneous tissue is not necessary, which eliminates skin-related complications by preserving subcutaneous circulation, resulting in less intra-operative blood loss, a shorter recovery time, and consequentially, a decreased length of hospital stay. The skin-related complication rates were 11–24% in previous studies [7, 8]. This was the main reason that we had chosen ECST for our patient instead of conventional CST.

In the previous papers reporting ECST for adult patients [5, 7, 9], the first port was placed from the subcostal incision, and the balloon trocar was used for creating the space between the external and internal oblique muscles. Another one or two trocars were inserted in the space as working ports. We first created a small incision at the inguinal region and entered in the correct space between the external and internal oblique muscles without any difficulty, as it is commonly used for inguinal hernia repairs. Two ports were sufficient to release the external oblique muscle because the space created in infants was too small for three ports. Insufflation pressure helps in the blunt dissection of the space, and the balloon trocar was not necessary.

The space was spontaneously widened as the transection of aponeurosis proceeded. However, caution must be exercised to not dwell on one point for a prolonged period because it would result in burns to the skin since the fat tissue is very thin in infants. It would be extremely important to recognize that the aponeurosis is a thin membranous tissue, so that a little duration would be enough for its transection.

Furthermore, we used a Vicryl® mesh sheet as onlay and underlay to reduce the tension of the midline closure of the rectus abdominis muscles, which might not have been necessary if the abdominal wall were slid enough and could be closed without tension. However, ECST has been reported to have a similar recurrence rate as open CST [7, 8], thus, Slater et al. [10] recommended mesh augmentation. In this patient with a giant ventral hernia, we expected that the abdominal wall after CST should be thinner and the intra-abdominal pressure would be relatively high because of the smaller abdominal capacity, so we were worried that there could be some possibility of midline wound dehiscence followed by the recurrence of hernia. The absorbable Vicryl® mesh is considered useful to diminish prosthetic-related infection and minimize the risk of recurrence.

To the best of our knowledge, this is the first report of ECST performed on an infant with a giant abdominal wall defect. The technique was simple and safe, and results obtained were satisfactory.

## Conclusions

We present a case of giant ventral hernia after staged surgery for a large omphalocele. A large abdominal wall defect of the patient was successfully closed by ECST. ECST was relatively simple and safe for infants. And the cosmetic results were satisfactory.

## Abbreviations

CST: Component separation technique
ECST: Endoscopic component separation technique
